# House ownership, frequency of illness, fathers’ education: the most significant socio-demographic determinants of poor nutritional status in adolescent girls from low income households of Lahore, Pakistan

**DOI:** 10.1186/s12939-017-0621-z

**Published:** 2017-07-11

**Authors:** Fatima Hassan, Muhammad Asim, Shafya Salim, Ayesha Humayun

**Affiliations:** 1 Department of Higher Education, Punjab, Pakistan; 2 Statistical officer NHRC,PHRC Research Center, Shaikh Zayed Medical Complex, Lahore, Pakistan; 3 Government College of Home Economics, Gulberg, Lahore, Pakistan; 4 Department of Public Health and Community Medicine & Deputy Director at Department of Undergraduate Medical Education, Shaikh Khalifa Bin Zayed Al-Nahyan Medical College and Shaikh Zayed Post Graduate Medical Institute, Shaikh Zayed Medical Complex, Lahore, Pakistan

**Keywords:** Nutritional status, Adolescent girls, BMI, Socio demographic factors

## Abstract

**Background:**

Socio demographic factors besides dietary factors play important role in determining the health status of an individual. Health and nutritional Intervention programs stand a greater chance of success if planned, keeping the socio demographic characteristics of a certain population in focus. The present study was conducted to identify those socio demographic determinants which have a significant association with poor nutritional status in adolescent girls belonging to economically deprived households of Lahore.

**Methods:**

A cross-sectional analytical study of 140 adolescent girls living in the peri urban communities of Lahore was conducted. Socioeconomic and demographic data of the participants was recorded through a pretested questionnaire. Body mass index (BMI), a commonly used anthropometric measurement was taken as an indicator of nutritional status. Below normal (<18.5 m2) BMI was considered a benchmark of malnutrition in adolescent girls. Frequencies and percentages for socio demographic variables were calculated and Fisher Exact test was used to find out the association of nutritional status with socio-demographic predictors. Stepwise backward logistic regression analysis was then run to identify the most significant determinants associated with poor nutritional status in the adolescents. *P* < 0.05 was considered statistically significant.

**Results:**

Chronic energy deficiency was highly prevalent among adolescent girls, 58% had BMI < 18.5 m2. Binary regression analysis result showed that the participants who lived in rented houses (AOR = 3.513; 95% CI = 1.366 to 9.031) who fell ill frequently (AOR = 2.996; 95% CI = 1.198 to 7.491) whose fathers were illiterate (AOR = 2.941; 95% CI = 1.187 to 7.287) were at greater odds of having poor nutritional status. Interestingly, the participants who lived in joint families (AOR = 0.411; 95% CI = 0.145 to 1.168) and were more frequently food insecure (AOR = 0.431; 95% CI = 0.164 to 1.133) had lesser odds of having poor nutritional status than those who lived in nuclear families and were food secure.

**Conclusion:**

Frequency of illness, house ownership and fathers’ education are the determinants positively associated with poor nutritional status of adolescent girls. Food insecurity and joint family structure were negatively associated with poor nutritional status. The study will help in planning interventions for improving nutritional status of adolescent girls by targeting the significant socio demographic determinants of poor nutritional status among this group.

## Background

Socioeconomic status (SES) can be broadly conceptualized as one’s position in the social structure. Sociologists emphasize a Weberian approach that encompasses the notions of class, status, and power [1]. Socio economic status is determined by certain socio demographic factors such as housing characteristics, number of persons living in a room, food security, household income, occupation of the family, education level, and employment status of the person and access to health facilities and hygiene practices. The studies carried out in the past have used these predictors to determine the SES of a person. The influence of SES on health and consequently nutritional status is assumed to begin early in life, perhaps even in the prenatal environment, and continue to accumulate throughout life. SES is thus more than financial well-being or educational achievement; it encompasses a lifetime of access to knowledge, resources, and opportunities [2, 3]. A large and growing body of evidence shows those socio demographic factors – age, race, ethnicity, and language, for example – and socioeconomic status (SES), such as income and education, can influence health and nutritional outcomes [4]. Ever-increasing evidence suggests that the health and nutritional status of a population is greatly determined by the social and economic circumstances of that population, as well as its access to health care services [5–7].

### Housing and family characteristics

The type of dwelling also to a large extent determines socio economic status one belongs to. It has generally been seen that poor people tend to live in overcrowded dwellings or more people are sharing a single room as compared their more affluent counterparts. Previous studies have also shown that poverty is associated with poor and crowded housing and stressful working conditions [5, 8].

### Food security and gender equality

Another area where people with Low SES compromise a lot is food. Low income not only reduces purchasing power of a family but it also limits the food choices one can make, especially resulting in cutting down on nutritionally high quality food such as meat, milk and fruits. This leads to food insecurity. Food security not only means “to have enough to eat” but also “to have nutritionally adequate diet”. Most people belonging to low socio economic status families face the problem of not eating quality diet which is also inadequate in terms of nutrients while a few also experience the issue of quantity as well. Being food secure also means the excess of population to food markets and food stores [9]. Similarly gender issues are quite common in South Asia where people still give preference to boys and male members of the family and women are supposed to eat later than men. Sometimes the girls in the family are also served comparatively lesser portion, quantity and quality wise as compared to their brothers. Physical growth of adolescent girls’ is related to their dietary intake which is determined by availability of food in terms of quality and quantity and the ability to digest absorb and utilize food. Food availability is influenced by dietary practices, cultural traditions, family structure, birth intervals, meal patterns, and food allocation.

One of the most important factors which determine the food choices one can make is the purchasing power of that person. It in a way also determines how much can one spend on buying food. A financial survey carried out in the recent past reported that per capita income has increased from $1513 in 2014–15 [10]. The report does not take into account the inflation which has increased multifold thus reducing the purchasing power of a person and as reported in previous studies that with chronic poverty, a process called with chronic poverty “Engel’s phenomenon” occurs. Food selection narrows down to those items providing the most energy at lowest cost.

“Over time, micronutrients disappear from the diet, and specific nutrient deficiencies follow. For families living well below the poverty level, increasing income does increase discretionary spending. Any money received is used to pay the cost of necessities- food, rent, heat and the expenses such as clothes and transportation”. Nothing is left to improve the nutritional quality of diet. Impoverishment increases the micro nutrient deficiencies. Discretionary income refers to the fund available after obtaining necessities. These costs include food, housing, health care, clothes and transportation [11]. Furthermore it was found in a study that, women who are socio economically disadvantaged experience more stressful life events and more chronic stress as compare to socially and economically well placed women [12, 13].

### Education and health behavior

Education and income as important socio demographic variable have always been a focus of researchers investigating association of important demographic factors with health outcomes. Previous studies have also shown considerable international evidence that education is strongly linked to health and to determinants of health such as health behaviors which also includes making healthier eating choices [4]. Level of education also seems to be an important predictor of nutritional status possibly because of two reasons. First with a higher level of education one tends to get a better paid job hence also enhancing the purchasing power of that person so that one can spend more on buying quality food. Secondly, better education might also mean making nutritionally wise choices hence improving the nutritional status. The studies in the past have found a significant association between education level and literacy of the fathers and adolescent girls as the teenagers whose father had a higher level of education had better nutritional status than the girls whose fathers were illiterate or had lower level of education [2]. Higher education provides explicit facts, and leads to attitudes and behaviors that are conducive to better health as well as a willingness to delay gratification in order to achieve desired goals. Persons of higher status smoke less, eat better, and exercise more than persons with fewer resources [14, 15].

### Health and hygiene

The economists have marked the availability of health facility to population as an important determinant of socio economic status. Poor public and private health services may have direct and indirect impacts on an individual’s health by limiting access to quality health care, healthy foods, and recreational opportunities [16, 17].

While access to health facilities such as hospitals and medical care centers might be seen as a state responsibility, there are certain other criteria which determine one’s access to health and hygiene at individual level. Availability of water and sanitation facilities such as toilets and washrooms come under such criteria. It determines to a large extent, one’s access towards basic personal hygiene such as bathing.

### Working status of the adolescent girls

One of the major predictor of socio economic status is the working status of the adolescent girls of a family as usually it is a norm in South Asia that children are financially supported by their parents even well into their early adult life until they get a job. This also holds true with the adolescents especially the girls, belonging to high or middle income groups as they do not work, to support their education or families. The adolescent girls who belong to low socio economic status often have to step outside their homes to economically support their families. A study carried out in 40 countries around the world on adolescent girls found out the higher prevalence rates of moderate and severe thinness in Asia [18]. The studies carried out in the past have found that adolescent girls were mostly illiterate as they had to leave school to financially support their families [19]. This usually results in compromised health and nutritional status of these working adolescent girls and they are more likely to suffer from different nutritional deficiency diseases especially iron deficiency anemia. A study carried out on female garment factory workers in Bangladesh found out that these working girls suffered from different nutritional deficiencies especially a high prevalence of anemia was observed [20].

In similar studies carried out in the past high work participation by the young teenage girls was reported to be one of the contributing factor towards higher under nutrition in these girls [2].

Health according to WHO is been defined as a state of complete well being of an individual and not mere absence or presence of disease [21]. Similarly nutritional status of an individual depends on a lot of food and nonfood factors, eating healthy, staying active, having good health, access to education, hygiene habits, state of food security, household budget etc.

### Scenario in Pakistan’s perspective

Pakistan is a densely populated country with 5th largest population of 20 billion in the world. With largely unequal distribution of wealth, and lack of priority given to health sector on the part of government, malnourishment is rampant among those who are poor. A vicious cycle of poverty and malnourishment often results in poor health and nutritional status of the population. Whereas nearly all population groups are affected by this state of affair, the situation becomes more worrisome when this malnutrition is masked behind a growth spurt of adolescent years and only manifests itself when these chronically malnourished adolescent girls enter into womanhood and start bearing children. The result is, Pakistan has one of the highest maternal mortality and Low Birth Weight [LBW] ratios in the world [22]. National Nutrition Survey [NNS] 2011 has also shown that nearly half of Pakistani women are suffering from chronic malnutrition. Thus malnutrition must be addressed in adolescence, taking the life cycle approach, before the vicious cycle is repeated in children [23].

Whereas a lot of effort on the part of government in particular and community in general is required to realize the importance of adolescent girl nutrition as a future investment into healthier mothers and nation, the steps also need to be taken to identify those socio demographic determinants which are most significantly associated with poor nutritional status of adolescent girls belonging to low income families. Therefore this study was undertaken to identify those socio demographic factors which are significantly associated with poor nutritional status of adolescent girls.

### Methods

A cross sectional analytical study was conducted between August 2013 to February 2014 at *Shah di Khoee* and *Mochipura*, both are urban slum communities in Lahore. These communities were selected through convenience sampling (geographical accessibility). All adolescent girls residing in these urban slums were our study population and the participants were recruited with the help of Lady Health workers of both areas. As no prevalence study in context to BMI of adolescent girls was available, sample size was calculated on the basis of anemia prevalence among adolescent girls belonging to low income group in Pakistan which was found to be above 93% in early adolescents (14 years to 16 years) and 60% in late adolescents (17 years to 19 years), taking mean of these two groups, prevalence among adolescent girls was taken as 76.5% [24]. Using single proportion estimation, confidence level at 0.95 and margin of error at 7%, a sample size of 140 was calculated.

BMI was taken as a benchmark of nutritional status indicator. BMI less than 18.5 m2 was judged as an indicator of under nutrition. Similarly girls having BMI more than 24.9 m2 were identified as overweight or obese. Adolescent girls who had BMI below or above normal (18.5–24.9 m2) were identified as having poor nutritional status.

The research was conducted in compliance with the ethical principles for medical research involving human subjects of the Helsinki Declaration. Verbal informed consent for interview was taken from all participants and their parents as well, in front of a witness. The right to privacy, anonymity, voluntary participation and confidentiality were observed.

### Data collection

#### Anthropometric measurements

Height and weight of the girls were measured. Height was taken in cm using a wooden board fixed with a plastic tape to the nearest 0.1 cm. Beurer scale was used to measure Weight in kilograms. BMI was calculated as the weight in kg divided by the square of the height in meter and cutoff point of 18.5–24.9 m2 was used to distinguish normal BMI from below and above normal.

#### Socioeconomic, demographic and health data

Data was collected using a structured pre-tested interview guide. Socioeconomic, demographic and health information was collected on family and housing characteristics, age, participants working status and education, parental education, health status and hygiene practices, food security and gender equality.

Descriptive statistics were used along with fisher exact test and Independent sample t-test for comparison of means in two groups. Final analysis was done on 112 forms due to incomplete information in 18 forms and damage to 10 forms. Statistical analysis was carried out using Statistical Package for the Social Sciences (SPSS v.17).

## Results

### Housing and family characteristics

The frequencies and percentages were calculated for these determinants and the results showed that 59 (52.7%) families were living in a single room house and 82 (73.2%) of the girls’ families were using only one washroom. About 59 (52%) of the participants were living in family owned houses as opposed to 45 (40.2%) who were living in rented accommodation. Sixty five (58%) participants’ fathers were working as laborers or on daily wages.

All the subjects belonged to low income group. Eighty four (77%) participants’ family comprised of 5–8 family members and 24 participants were first born (24.1%). The study might be seen pointing towards a big cultural change in urban and semi urban areas with 81 (72.3%) reporting to be living in nuclear families as compare to just31 (27.7%) living in joint family system.

### Family income and expenditures

In most of the households 91 (81.3%) 1–3 family members were contributing towards total family income. More than 40% of the girls’ families had income below ten thousands rupees whereas 33% had monthly income between eleven to fifteen thousand rupees. A mere 5% had income above thirty thousand as around three or four people were working in these households. Almost a quarter of families (25%) had per capita income below half a dollar that is 55 rupees in local currency. Household bills, mainly the electricity bills constituted the main expenditure 73 (65.2%) followed by the rent 24 (21.43%). (Table 1).

**Table 1:** Housing and Family Characteristics

Variable	Level	Frequency	Percentage	Fisher Exact value	*P*- value
No. of rooms in the house	1	59	52.7	2.668	.085
	2	25	22.3		
	3–4	28	25		
No of washrooms	1	82	73.2	2.859	.083
	2	26	23.2		
	3	4	3.6		
Ownership of the home	Owned by the family	59	52.7	6.683	.007
	Rented	45	40.2		
	other	8	7.1		
Occupation of the head of family	Laborer	65	58	3.435	.053
	Office Worker	14	12.5		
	Salesman	11	9.8		
	Driver	11	9.8		
	Other	11	9.8		
No. of family members at home	Upto4	3	2.7	1.772	.153
	5–8	84	77		
	9–12	22	21.3		
Ordinal position among siblings	1	27	24.1	-	-
	2	22	19.6		
	3	18	16.1		
	4	22	19.6		
	5	11	9.8		
	6	6	5.4		
	7	4	3.6		
	9	2	1.8		
Type of family structure	Nuclear	81	72.3%	.417	.521
	Joint	31	27.7%		
Number Of Working Family Members	1	29	25.9	1.038	.275
	2	32	28.6		
	3	30	26.8		
	4	16	14.3		
	5	5	4.5		
Average household income/month	Rs 4000–12,000	31	27.7%	2.480	.092
	Rs13000–15,000	32	30.3%		
	Rs 16,000–20,000	25	22.4%		
	Rs 25,000–60,000	22	19.6%		
Per capita income	Up to Rs54/day	27	25	.157	.684
	Up to Rs80/day	24	22.3		
	Up to Rs 100/day	32	28.6		
	Up to Rs 167/day	27	24.1		
Expenditure other than food	Bills	73	65.2	.121	.689
	Rent	24	21.4		
	Medicines	6	5.4		
	Education fees	2	1.8		
	Other	7	6.3		

### Personal and Educational characteristics of the participants

51 (45.5%) participants were between the ages of 13–15 whereas 61 (54.5%) were between the ages of 16–19. Similarly 48 (42.9%) were currently in education as opposed to 64 (57.1%) of which 29 (25.9%) had never been to a school and were illiterate. Most of the participants’ father 50 (44.6%) were illiterate and 27 (24.1%) had education up to primary level. Similarly 73 (65.2%) mothers of the participants were not educated and 24 (21.4%) had education upto primary level (Table 2).

**Table 2:** Personal and Educational Characteristics of the Participants

Variable	Level	Frequency	Percentage	Fisher Exact value	*P*- value
Age of Participant	13–15	51	45.5	1.245	.249
	16–19	61	54.5		
Currently in education	Yes	48	42.9	2.542	.087
	No	64	57.1		
Highest level of education of participant	Class 5	31	27.7	6.078	.009
	Class 8	25	22.3		
	Class 10	20	17.9		
	Intermediate	6	5.4		
	Masters	1	.9		
	Not educated	29	25.9		
Highest level of participant’s father’s education	Class 5	27	24.1	8.305	.002
	Class 8	14	12.5		
	Class 10	19	17.0		
	Intermediate	1	.9		
	Masters	1	.9		
	Not educated	50	44.6		
Highest level of participant’s mother’s education	Class 5	24	21.4	6.078	.009
	Class 8	8	7.1		
	Class 10	4	3.6		
	Bachelors	3	2.7		
	Not educated	73	65.2		

### Health status and hygiene practices of the participants

A majority 65 (58%) of the adolescents had low BMI than normal and only a small number of 9(8%) were found to be overweight. Eighty seven participants (77.7%) reported to be born healthy as opposed to 25 (22.3%) who were born weak. Sixty five (58%) participant reported a history of illness in the past 6 months with chest and throat infection being the main reason at 24 (21.4%). General health of the participants was found to be poor with 60 (53.6%) of the participants reporting a shortness of breath and lethargy both. Thirty eight (33.9%) adolescent girls also reported to be weekly falling ill.

Similarly 28 (25%) reported to take baths weekly as compared to 44 (39.35) and 39 (34.8%) adolescents who reported of taking baths twice and thrice a week respectively. (It must be kept in mind that the data was collected during winters). There seemed to be a healthy reproductive health with 103 (92%) reporting to have regular menstruation cycle.

Eighty six (76.8%) reported using cloth as a sanitary napkin during special days (mensturation period) of the month with 50 (44.6%) reporting of reuse of cloth napkin after washing with soap and water (Table 3).

**Table 3:** Health Status and Hygiene Practices of the Participants

Variable	Level	Frequency	Percentage	Fisher Exact value	*P*- value
BMI	Normal	38	33.9	2.8	.26
	Underweight	65	58.0		
	Overweight	9	8.0		
Health status at birth	Healthy	87	77.7	.208	.646
	Weak	25	22.3		
History of illness over the past 6 months	Yes	65	58.0	.475	.440
	No	47	42.0		
Details of illness that occurred in the past 6 months	Dengue	12	10.7	-	-
	Gastro Intestinal Tract	12	10.7		
	Throat and Chest	24	21.4		
	Other	17	15.1		
	not fallen ill	47	42.0		
Frequency of illness	Weekly	38	33.9	7.024	.005
	Monthly	36	32.1		
	Twice a year	38	33.9		
General health	Lethargy	23	20.5	.475	.440
	Shortness of breath	12	10.7		
	Both	60	53.6		
	Neither	17	15.2		
Frequency of baths	Weekly	28	25.0	3.549	.047
	Twice a week	44	39.3		
	Thrice a week	39	34.8		
	Daily	1	.9		
Regularity of periods	Yes	103	92.0	.000	1.000
	No	9	8.0		
Type of sanitary napkin usage	Cloth	86	76.8	.885	.255
	Pad	14	12.5		
	Both	12	10.7		
Reuse of sanitary napkin	No	62	55.4	6.233	.012
	Yes	50	44.6		
How sanitary napkin is cleaned	Wash with Soap	50	44.6	-	-
	Wash without soap	3	2.7		
	Does not reuse	59	52.7		

### Food security and gender equality

A vast majority 81 (72.3%) girls reported that food they bought did not last the whole month. Thirty eight (33.9%) reported that their families experience food shortage every month. Society remained mostly patriarchal with 83 (74.1%) reporting fathers to be the main figure who are first served meals. Gender bias was not found with an overwhelming majority of 106 (94.6%) reported to be served the same food as that of their brothers. Similarly 89 (79.5%) reported that their families saved food for them when they were at work or tuition (Table 4).

**Table 4:** Food Security and Gender Equality

Variable	Level	Frequency	Percentage	Fisher Exact value	*P*- value
Food bought not lasting	Yes	81	72.3	1.136	.209
	No	31	27.7		
Frequency of food shortage	Every month	38	33.9	.050	.690
	Some months	42	37.5		
	Never	32	28.6		
Who is served meals first in the family	Father	83	74.1	-	-
	Mother	16	14.3		
	Brother	10	8.9		
	Sister	1	.9		
	You	2	1.8		
Are you served the same food as your brothers	Yes	106	94.6	.000	.694
	No	6	5.4		
Is food saved for you if you skip meals	Yes	89	79.5	.415	.465
	No	21	18.8		
	Doesn’t go out	1	.9		
	sometimes but not always	1	.9		

Binary logistic regression results identified house ownership (AOR = 3.513), frequency of illness (AOR = 2.996) and fathers' education of the participants (AOR = 2.941) as the most significantly associated social determinants of poor nutritional status among these girls. Whereas, frequency of food shortage (AOR = 0.431) and living in a joint family (AOR = 0.411) were negatively associated with poor nutritional status with odds of having poor nutritional status (Tables 5 and 6).

**Table 5:** Binary logistic regression analysis for the association of different variables with below normal BMI among adolescent girls

Variable	OR	95% C.I.for EXP(B)		AOR	95% C.I for EXP(B)		
		Lower	Upper		Lower	Upper	Sig.
Work status	2.48	1.147	5.380	2.062	.284	14.990	.475
Student or not	2.000	0.931	4.297	.125	.016	.962	.046
Menstruation period regularity	0.896	0.227	3.532	.787	.098	6.309	.822
Frequency of illness	3.194	1.418	7.198	8.028	1.959	32.900	.004
Age category	1.661	.775	3.564	1.495	.529	4.224	.448
Number of rooms	2.025	.945	4.339	.349	.071	1.712	.194
Number of washrooms	2.267	.968	5.306	1.465	.301	7.127	.636
House ownership	3.002	1.367	6.596	6.103	1.610	23.132	.008
Fathers’ occupation	2.221	1.029	4.795	2.796	.814	9.604	.103
Earning members of family	.586	.250	1.373	1.024	.197	5.326	.978
Average household income	2.379	.919	6.156	4.791	.791	29.031	.088
Expenses other than food	.801	.362	1.771	.846	.218	3.280	.809
Transport expenditure	.376	.126	1.122	.287	.039	2.137	.223
Food insecurity	1.720	.747	3.965	1.859	.450	7.672	.392
Frequency of food shortage	.842	.382	1.856	.166	.036	.765	.021
Served same food	.710	.137	3.682	1.772	.118	26.524	.679
Food saved for the participant	1.569	.579	4.252	.968	.202	4.637	.968
Fathers’ education	3.456	1.544	7.737	2.282	.664	7.846	.191
Mothers’ education	2.935	1.314	6.554	.802	.206	3.122	.750
Health status at birth	1.379	.549	3.460	1.109	.235	5.243	.896
Six months health history	1.408	.658	3.011	.325	.076	1.382	.128
Type of family structure	.688	.292	1.619	.120	.023	.616	.011
Sanitary napkin	.496	.160	1.540	1.099	.191	6.330	.916
Reuse of sanitary napkin	.342	.155	.756	.532	.082	3.442	.508
Bath frequency	2.298	1.043	5.061	2.071	.569	7.534	.269
Per capita income	.784	.354	1.736	.162	.030	.879	.035
Food expenditure out of total budget	.469	.179	1.227	.550	.101	2.981	.488

**Table 6:** Final model of bivariate regression showing association of most significant variables with below normal BMI among adolescent girls

Variable	OR	95% C.I.for EXP(B)		AOR	95% C.I for EXP(B)		
		Lower	Upper		Lower	Upper	Sig.
Frequency of illness	3.194	1.418	7.198	2.996	1.198	7.491	.019
House ownership	3.002	1.367	6.596	3.513	1.366	9.031	.009
Average household income	2.379	.919	6.156	2.901	.975	8.630	.055
Frequency of food shortage	.842	.382	1.856	.431	.164	1.133	.088
Fathers’ education	3.456	1.544	7.737	2.941	1.187	7.287	.020
Type of Family structure	.688	.292	1.619	.411	.145	1.168	.095
Constant				470.844			.003

## Discussion

### Housing and family characteristics

Economists have often used the factor of crowding as an indicator of wealth status. Those who belong to poor households or have less income tend to have more crowding factor than those who are economically better. Past studies also identify household size, persons/room and land holding are significant determinants of socio economic status [2, 25].

Same was found in the present study as it was observed that 52.7% of the adolescents were living in one room house and moreover 73.2% had one washroom for the whole family. In South Asian culture, a great importance is placed on material assets such as land and cattle ownership. Whereas land and cattle ownership is given importance in rural settings, in cities it is usually ownership of the house, along with the locality it is situated which determines one’s social status. A family who does not own a house is generally considered poor. Physical assets are given importance throughout the world but in some cultures a great importance is placed on land and cattle ownership as they are considered a status of wealth and respect [26]. In the present study it was found that around 40% of the adolescent families were living in rented houses and further analysis found house ownership as one of the most significant determinant of poor nutritional status. It might be due to the fact that as major part of income is gone on paying rent, compromise has to be sought on buying nutritious food. The present study is in consistent with the results of a Canadian study which also identified not having house ownership as a reason of being food insecure [27]. However, the present study showed no significant association between family size and nutritional status. This finding again is in line with findings of previous studies which showed no significant association between family size and nutritional status [2].

### Family income and expenditures

As the study participants also included working adolescents, number of family members who were employed ranged from 1 to 5, with 25% families having one employed member to 28.6% and 26.8% families having 2 and 3 employed members respectively. Average household income showed more than a one fourth (27.7%) adolescent families as having income that ranged between 4000 to 12,000 rupees/month($1 = Rs 100 at the time of data collection). About 30.3% and 22.4% families had income between 13,000–15,000 and 16,000–20,000 respectively. Per capita income was calculated and it was found that 25% adolescent girls’ family members were living on less than 55 rupees/day/person which makes it around half cent/day. Whereas 28% families were living on Rs 100/day/person. The findings of the study were not in consistent with previous studies which showed no significant association (*p* > 0.05) between per capita income and nutritional status of the participants [2]. Average household income was also found to be one of the significant determinant of poor nutritional status in final regression model with AOR = 2.901.

Household bills which on probe was found to be mostly of electricity took the major chunk of poor household incomes as 65% reported it to be the next major expenditure after food, with rent resuming the second place at 21.4%. Studies in the past have shown that the type of housing one lives in for example rented, mortgaged, housing instability, struggle with mortgage or rent payments/behind on rent, moved to a different dwelling for cost or other such arrangements also have negative implications on health status of an individual [25, 28].

About 58% of the adolescents’ fathers were working as laborers indicating towards lack of skills and education in poor households and consequently low incomes. The studies have also shown that occupation of the family is strongly related with the risk of under nutrition in adolescent girls as mal nutrition was found to be more prevalent in families whose occupation was labor as compared to the girls who belonged to families whose main occupation was business [2].

An increasing trend towards nuclear families was witnessed as more than 72% adolescents were living in nuclear families. This trend is in clash with traditions of South Asian culture where a great value is placed on joint family systems and elders such as grandparents and paternal uncles are given a lot of importance. A later regression analysis also supported joint family system which showed that girls who lived in joint families had lesser odds of being malnourished than those who lived in nuclear families. It shows that joint families have a protective effect towards malnourishment. It might be due to the reason that grandparents being comparatively free of other stresses of work spend more time with their grand children and are better able to take care that their grand children have eaten enough. Another plausable explanation is that in joint families, more food choices might be available as there are more persons to contribute towards family income thereby increasing purchasing power. Further research on this aspect is needed to confirm these hypotheses.

### Personal and educational characteristics of the participants

The adolescent girls were divided into two groups according to age, the early adolescents (13 years to 15 years) and late adolescents (16 years to 19 years). It was found that there existed no significant association between age and BMI (>0.05). The findings of this study were not in consistent with previous study findings which showed a significant association between nutritional status and age [2]. However, the present study showed significant association between education of the participant and their nutritional status (< 0.05). This finding is in line with previous studies which show an improved nutritional status with increase in education level [2, 29]. Similarly the study showed significant association between education of fathers and nutritional status of the participants which is in consistent with findings of previous study which showed a decline in malnutrition with increase in fathers’ education [2]. This difference might be due to the fact that with increase in education one is more likely to get a better paid job which automatically increases the amount one tend to spend on food budget. The results of this study were also consistent with other studies which showed significant association between nutritional status of the participants and their mothers, education level [30, 31].

A recent study in India has also showed a strong correlation between the nutritive food intake by adolescent girl and education of the fathers [32]. A study carried out in India with same socio economic setting has also found literacy status of father to be significantly associated with the nutritional status of the adolescent girls [2].

### Health status and hygiene practices of the participants

58% of the participants were found to underweight with BMI < 18.5 m2, whereas 33.9% were falling in the normal category with BMI 18.5–24.9 m2.Only a small percentage of 8% fell in overweight BMI > 25 m2. This finding is in consistent with previous findings that malnourished girls had low BMI [2, 33].

The previous studies also showed similar findings as the nutritional status of female garment factory workers in Bangladesh was found to be poorer [20]. Whereas past studies have established a link between adult obesity and high birth weight [34] however no association between health status at birth and low BMI was found as nearly 78% participants reported that they were born healthy and of normal weight.

Morbidity has long been associated with ill health. A past study has also shown the vicious cycle of poor health and frequently recurring infections [35]. General health was found to be poorer and it points towards vicious cycle of ill health and disease as 58% reported of falling ill within the past 6 months. Around 34% and 32% of the participants reported of falling ill weekly and monthly, thereby identifying frequency of illness as one of the most significant determinant of nutritional status (*p* < 0.05).

It was found that a majority of adolescent girls were taking bath only once a week, which might also be due to the fact that 73% of adolescent families had only one washroom for the family as the economist agree that Socio economic status also determines to a large extent the availability and access to proper sanitation which include not only safe drinking water but also opportunity to bath and toilet facility. In the past a study has also suggested that poor personal hygiene may contribute to the phenomenon of under-nutrition [2].

### Food security and gender equality

An overwhelming majority of around 72% said that their families become food insecure, of which around 34% experience it every month while 38% said food insecurity is experienced sometimes. Final regression model also found food insecurity to be one of the most significant determinant of nutritional status as it was found that girls whose families are food insecure have lesser chances of having poor BMI than those whose families are food secure, this finding is very different and might be due to fact that the girls who are food insecure tend to eat with more care than others for fear of not having enough to eat. However further research in this aspect is needed before a concluding statement can be given.

Gender bias was not found in the present study as nearly 95% girls reported that they are served the same food as their brothers and 80% reported that food is saved for them if they are not home. This finding is not in line with the finding of a previous study in Bangladesh which reported otherwise [36, 37].

The present study tries to identify the most significant socio demographic characteristics which are associated with poor nutritional status in adolescent girls belonging to economically deprived households. Although a few studies have been conducted in the past on adolescent girls but none of the study was community based according to the knowledge of this author. The study is especially important from the perspective that socio demographic determinants were studied. However, as the study was carried out on small scale due to financial, cultural and time constraints on the part of researcher, the results cannot be generalized to all the working adolescents. Nevertheless, this descriptive analytical study provides a new dimension for future studies in the same socio economic and geographical settings on a larger scale.

## Conclusion

It can be concluded from this study that certain socio demographic determinants such as house ownership, frequency of illness and fathers’ education are significantly associated with the poor nutritional status of adolescent girls belonging to low income households. The study also shows some interesting findings when it is found that living in joint families and being food insecure is negatively associated with poor nutritional status among adolescent girls. The findings of the present study can be used as a pilot study and future study on a larger sample throughout the country is recommended. This will help in drawing attention of the world towards an important role, socio demographic determinants play in health and nutrition of the population especially the adolescent girls who are future mothers. It can help in planning effective nutritional interventions for adolescent girls by targeting the most significantly associated socio demographic determinants of nutritional status in this age and socio economic group.

## Abbreviations

BMI: Body Mass Index
SES: Socio Economic Status.
